# Sex-dependent shifts in visual detection thresholds under turbid conditions in an African cichlid

**DOI:** 10.1093/conphys/coaf046

**Published:** 2025-07-14

**Authors:** J H Tiarks, S M Gray

**Affiliations:** School of Environment and Natural Resources, The Ohio State University, 210 Kottman Hall, 2021 Coffey Road, Columbus, OH 43210, USA; School of Environment and Natural Resources, The Ohio State University, 210 Kottman Hall, 2021 Coffey Road, Columbus, OH 43210, USA; Department of Biology, University of Prince Edward Island, 434 Duffy Science Centre, 550 University Ave, Charlottetown, PE C1A 4P3, Canada

**Keywords:** Contrast, Lake Victoria, optomotor, plasticity, vision, water quality

## Abstract

Turbidity is increasing in freshwaters globally due to human activities and is known to affect visually mediated behaviours in fish. As anthropogenic impacts continue to degrade aquatic environments, it is critical to determine how sensory systems are affected and what this might mean for population persistence. We investigated the effect of turbidity on visual detection thresholds in an African cichlid fish (*Pseudocrenilabrus multicolor*) that experiences environmental extremes across its East African range. We tested the visual abilities of adult wild-caught fish from two sites representing the extremes of turbidity and oxygen (a high turbidity, high dissolved oxygen river and a low turbidity, low dissolved oxygen swamp). Further, we reared offspring of wild-caught parents from each population in a full-factorial high/low oxygen, high/low turbidity design to tease apart the influence of each stressor on visual detection thresholds. We used an optomotor response test to determine detection thresholds under increasing levels of turbidity for both wild-caught and lab-reared fish. Detection thresholds were higher in the wild-caught river population compared to the swamp population, and there was a strong sex difference, such that wild-caught males had higher detection thresholds than females regardless of population of origin. Our results suggest that there are sex-based differences in contrast detection abilities that could play a critical role in visual ecology for populations experiencing divergent turbidity regimes. In the rearing experiment, sex-based differences in detection thresholds were influenced by different aspects of the rearing treatment. Detection threshold varied significantly by oxygen-rearing treatment for males and by the interactive effects of oxygen and turbidity for females. This research improves our understanding of the effect of elevated turbidity on African cichlid vision and contributes to growing knowledge of how animals respond to environmental change.

## Introduction

The primary function of sensory systems in vertebrates is to meet the ecological needs of an organism (Utne-Palm, 2002; Cronin *et al.,* 2014). This, in part, is driven by the ability to detect stimuli against the ambient background. The process of vision (e.g. signal transmission, signal reception) is particularly important for behaviours dependent on visual signals, such as species recognition, predator–prey interactions and reproductive cues. Anthropogenic change can disrupt various aspects of the visual process resulting in changes in animal behaviour and the visual ecology within a system. For example, eutrophication and increased algal growth can change the visual environment for many aquatic organisms, resulting in reduced visibility and detection. In a study on three-spined sticklebacks (*Gasterosteus aculeatus*), males increased the time and energy spent on courtship under algal turbidity conditions, while females simultaneously increased time spent inspecting potential mates (Candolin *et al.,* 2007). While there are documented instances of how organisms respond to anthropogenic change, there is also a growing body of literature across taxa that examines how organisms can cope with anthropogenic changes in their visual landscape (e.g. phenotypic plasticity, behavioural flexibility). In one example, populations of European hedgehogs, *Erinaceus europaeus,* alter their nocturnal foraging patterns to avoid increased road light and noise; urban populations were more active after 12 am when human activity and traffic was significantly reduced (Dowding *et al.,* 2010). While humans alter the visual landscape in a variety of ways, behavioural flexibility may be imperative in determining the persistence of populations faced with anthropogenic change, such that organisms still meet their ecological needs despite altered sensory landscapes.

One way anthropogenic change alters visual landscapes in aquatic environments is through increasing turbidity, or suspended particles in the water column. This is occurring in freshwaters globally in association with deforestation, intensive agriculture practises and wetland removal (through draining and burning) (Reid *et al.,* 2019). Turbidity elevated above normal or historic levels acts as an environmental stressor that can affect the behaviour and physiology of many aquatic organisms (Gray, 2016) and cause shifts in visually guided behaviours (van der Sluijs *et al.,* 2011) that negatively affect ecological systems. For example, turbidity increases anti-predator behaviour in guppies (*Poecilia reticulata*) (Ehlman *et al.,* 2015) and alters behavioural interactions between predator (walleye, *Sander vitreus*) and prey (emerald shiner, *Notropis atherinoides*) in Lake Erie (Nieman and Gray, 2019). Further, Nieman *et al.* (2018) found decreased visual ability in walleye and emerald shiner under different types (organic, inorganic) of turbidity, and increased turbidity has been shown to negatively affect visual acuity, or an organisms’ ability to detect objects (Caves *et al.,* 2017). Turbidity also changed the behavioural response of an African cichlid, *Pseudocrenilabrus multicolor*, with males exhibiting more aggressive behaviours when competing in turbid water compared to clear water (Gray *et al.,* 2012). Collectively, these examples suggest turbidity can influence a number of visually mediated behaviours that are essential for population persistence.

Cichlid fish are largely dependent on vision for reproduction: in many species, males use vision to defend territories against other males and to detect and court females, while females use vision to determine the quality of and choose males that express colourful and elaborate courtship displays. Given this, females and males may be reliant on different aspects of vision to meet their ecological needs. Changes to the visual environment, especially in historically clear waters, can act as a strong selective agent in visually reliant fishes, resulting in both plastic and genetic modifications of fish visual systems. Within a species of cichlids (*Pundamilia nyererei*), females from turbid environments have shown weaker preferences for male nuptial coloration compared to females from clear environments; concurrently, male nuptial coloration varied between turbid and clear environments (Maan *et al.,* 2010). In another example with Lake Malawi cichlids, even short-term changes to the visual environment (e.g. plumes or pulses of turbidity) caused an immediate change in behaviour, such that males moved away from their territories where they typically display courting behaviours (Gray *et al.,* 2011). We therefore expect shifts in visually mediated behaviours, such as courtship or mate choice, to correlate with shifts in visual ability with changes in the visual landscape.

Visual sensitivity refers to an organism’s ability to detect contrast under different light intensities and colours (Lythgoe, 1980) and is crucial in visually mediated behaviours (e.g. detecting mates or predators). Physiological measures of visual sensitivity (i.e. quantum catch of photons per wavelength) are important for quantifying the potential sensory abilities of fish (Dalton *et al.,* 2010); however, behavioural methods are helpful in understanding the ecological relevance of potential differences in sensitivity that lead to different detection thresholds (Kröger *et al.,* 2003; Smith *et al.,* 2012; Nieman *et al.,* 2018). Due to an innate optokinetic response to follow a moving object, fish will follow the contrast created by a moving black and white screen rotating around a tank (Cronin *et al.,* 2014). A visual detection threshold is reached when the fish stops following the screen because it can no longer detect the contrast between the moving bars due to altered visual environment. Many studies use the optomotor test to experimentally capture visual sensitivity by incrementally reducing the intensity of light (Arnold, 1974) and sometimes under specific wavelength bands of the light spectrum (Maan *et al.,* 2006; Matsuo *et al.,* 2021); however, optomotor tests can also be adapted to test for ecologically relevant detection thresholds in the face of elevated turbidity. Instead of directly altering the intensity of light under different colour spectra, aliquots of a turbidity solution are added to the water column as the screen rotates outside of the tank (e.g. Nieman *et al.,* 2018). The visual detection threshold is inferred as the highest level of turbidity reached when the fish stops following the rotating screen and thus acts as a behavioural proxy of visual sensitivity (see Maan *et al.,* 2006; Nieman *et al.,* 2018).

*Pseudocrenilabrus multicolor* is a maternal mouthbrooding haplochromine cichlid with strong patterns of sexual dimorphism (McNeil *et al.,* 2016; Atkinson and Gray, 2022) and is found in diverse habitats (e.g. clear, tannin-stained swamps; flowing rivers and lakes (Chapman *et al.,* 2000)). For instance, measurements from one swamp site of 1.5 mg l^−1^ dissolved oxygen (mean monthly dissolved oxygen; DO) and 1.5 Nephelometric turbidity units (NTU) contrasts with measurements from a nearby river/lake site of 6.07 mg l^−1^ (mean monthly DO) and 12.3 NTU (turbidity) (Chapman *et al.,* 2000; Chapman *et al.,* 2002; McNeil *et al.,* 2016); yet *P. multicolor* inhabit both sites. Thus, *P. multicolor* found in these two habitats experience extremes of dissolved oxygen and turbidity. Male *P. multicolor* also exhibit differences in nuptial coloration between swamp and river populations that may relate to the visual landscape (McNeil *et al.,* 2016; Atkinson and Gray, 2022). For example, males from swamp populations tend to display darker (i.e. lower overall percent reflectance) and more red nuptial coloration, while river populations tend to be brighter (i.e. higher overall percent reflectance) and more yellow, potentially using the ambient backgrounds (i.e. tanin-stained swamp or diffuse orange rivers) to maximize contrast, respectively, to enhance detectability (Gray *et al.,* 2012; McNeil *et al.,* 2016). Additionally, there is significant divergence among populations in other traits such as gill, brain and body morphology associated with low DO (Chapman *et al.,* 2000; Chapman *et al.,* 2002b; Crispo and Chapman, 2010).

To determine the influence of exposure to elevated turbidity on the visual abilities of *P. multicolor*, we used a behavioural test of visual detection thresholds (i.e. optomotor) in adult wild-caught fish and F1 fish reared in a fully reciprocal rearing experiment under two oxygen (high, low) and two turbidity (high, low) conditions. Due to the importance of mate choice and sexual dimorphism in colouration, we predict there will be a difference in visual abilities for males and females. For wild-caught fish, we expect behaviourally expressed visual sensitivity to be affected by long-term exposure to turbidity, such that fish chronically exposed to turbidity (i.e. river population) will be better able to detect objects under higher levels of turbidity compared to fish not regularly exposed to turbidity (i.e. swamp population). We predict this trend will hold true in the rearing experiment (i.e. fish reared in turbid water will be able to better detect objects under higher turbidity compared to fish reared in clear water). We do not expect hypoxia to directly affect visual abilities, but indirect effects may occur, such as energy allocation towards or away from the visual sensory system (e.g. optic centres of the brain). Multiple stressors (e.g. combination of turbidity and hypoxia) may shape visual abilities (Wong and Candolin, 2015). Generally, organisms are expected to respond to the interaction of multiple stressors in one of three ways: additive (where the interaction is the sum of the individual stressor effects), synergistic (where the interaction is stronger than expected) or antagonistic (where the interaction is weaker than expected) (Hale *et al.,* 2017). Given this, we may expect *P. multicolor* reared in the combined effects of hypoxia and turbidity to have different visual capabilities than those reared under individual stressors or those from the wild.

## Materials and Methods

### Ethics statement

All research was approved by The Ohio State University Institutional Animal Care and Use Committee (2014A00000055-R1). Permission was given by the Commissioner of Fisheries Resources Management and Development of Uganda for export of fish and from the Uganda National Council for Science and Technology for permission to conduct research.

### Optomotor response trials

To determine if exposure to turbidity affects visual sensitivity in *P. multicolor*, optomotor response trials were used to examine visual sensitivity in wild-caught fish in the field and rearing experiment fish in the lab (see details, below). This method was adapted from similar optomotor studies (Maan *et al.,* 2006; Nieman *et al.,* 2018). A rotating black and white screen was placed outside a cylindrical tank filled with water (Cambro; 5.6 l; 23 × 19 cm; 9.5 cm radius). The screen was 5 mm from the outside of the tank to minimize viewing distance between the fish and screen. A baseline water clarity of ≤2.0 NTU was established in the cylindrical tank. After a 30-min acclimation in the cylindrical tank, the screen began to rotate (8 rotations/minute, bar width: 2.8 cm), initiating an optomotor response in the fish (i.e. swimming in the direction and at the speed of the rotating screen). If the fish did not show an optomotor response in the first 2 min, the trial was stopped, and the fish was not used. If an optomotor response was observed, we incrementally added 2.5 ml aliquot of turbidity slurry that increased turbidity in the tank by ~4 NTU per step every 2 min until the response was no longer observed (i.e. the fish stops following the screen). The turbidity slurry used consisted of a 1:4 ratio of fine sediment collected from the field sites to filtered water. The turbidity step, or level of turbidity (NTU), at which the fish stops swimming was considered the visual detection threshold and was the variable used for analysis. Due to the nature of the trials, a higher detection threshold (i.e. higher visual sensitivity) indicated that the individual fish could see better than a fish with a lower detection threshold (i.e. lower visual sensitivity). The optomotor response trial was repeated for each fish in both directions in a randomly determined order: once clockwise, once counterclockwise. We used the mean detection threshold of the clockwise and counterclockwise trials as our final value for analysis. Upon completion of both trials for an individual, we measured total length (cm), standard length (cm) and mass (g), recorded sex, population, rearing treatment (when applicable) and took a lateral view picture of the fish.

### Wild-caught fish

#### Description

Adult *P. multicolor* were collected in 2022 at two sites in the Lake Nabugao drainage basin, part of the Lake Victoria system of western Uganda. Sites were chosen to represent environmental extremes of turbidity and DO (Crispo and Chapman, 2010; McNeil *et al.,* 2016): Lwamunda (low turbidity, hypoxia; hereafter: swamp) and Ndyabusole (high turbidity, normoxia; hereafter: river). Standard baited minnow traps were set for 2–3 h and checked every 30 min. Fish were transported immediately to the Lake Nabugabo Field Site and held in lab conditions (e.g. lake water, low turbidity, normoxia) until trials were conducted (as described above; Table S1). In the field experiment, multiple steps were taken to control for lighting. All optomotor tests were conducted between 8 am and 5 pm under an awning. Thus, natural, ambient light was always used and tests were conducted under full shade. As an additional precaution, we used a diffusing screen to remove any potential glare or shadows.

#### Statistical analyses

To test if detection thresholds varied among wild-caught fish from different populations, we analysed the dataset using an analysis of covariance (ANCOVA) with mean detection threshold as the response variable, log10(standard length) as the covariate, and population and sex as fixed factors. Interaction terms that were not statistically significant (alpha ≤ 0.05) or that were not approaching significance (alpha≤0.1) were sequentially removed from the model until we arrived at the final model (Table S3). We tested all assumptions for parametric tests, and the appropriateness of ANCOVA was confirmed. Individuals were excluded from analysis if the difference between detection thresholds in Trial 1 and Trial 2 was ≥10 NTU (Fig. S1). We used an alpha ≤0.05 to infer a significant difference and alpha ≤0.1 to indicate a trend (Sullivan *et al.,* 2019). All statistical analyses were performed in R (R Core Team, 2024,), and all ANCOVAs were performed using *stats*.

### Rearing experiment fish

#### Description

As part of a larger project aimed at teasing apart the relative effects of DO and turbidity on trait differences in *P. multicolor*, we conducted a fully factorial split-brood rearing experiment in the laboratory (see details in Williams *et al.* (2024)). Briefly, wild-caught adult fish from swamp and river populations were transported to the Ohio State University (USA) in 2018 to serve as parental populations for the rearing experiment. We used two levels each of DO (<2 mg/l and 6–8 mg/l) and turbidity (<2 NTU and >10 NTU) such that we had four treatment combinations: normoxia/high turbidity, normoxia/low turbidity, hypoxia/high turbidity, hypoxia/low turbidity. One brood from each set of known parents (*n* = 10 parental pairs per population) was randomly split among the four treatment conditions and maintained under those conditions for >1 year. Optomotor assays were performed on mature, adult fish (Table S2).

#### Behavioural response trials

When fish reached maturity (>6 months), individuals were marked using Visible Implant Elastomer (VIE) tagging (i.e. black or white ink inserted under the skin in unique patterns and locations) so that they could be individually identified through various behavioural and physiological assays (Williams *et al.,* 2024). Individually identified fish underwent the optomotor response trials as described above. The turbidity slurry used consisted of a 1:4 ratio of fine bentonite clay to reverse osmosis deionization (RO/DI) water. The turbidity slurry used was the same turbidity slurry that was used during rearing to maintain experimental conditions in aquaria. The light environment in the lab setting consisted of broad-spectrum overhead lights (i.e. no UV); the lab environment allowed for more controlled and consistent lighting due to the nature of the environment.

#### Statistical analyses

Initially, linear mixed models (LMM) were performed for detection threshold with oxygen (hypoxic or normoxic), and turbidity (turbid or clear) rearing treatments, population of origin (swamp or river) and sex (female or male) as fixed factors. Brood was included as a random factor to account for relatedness between individuals and log10(standard length) was included as a covariate; however, brood was removed from the final model because it was not significant (likely because there were almost no replicates within broods). We then ran ANCOVAs, and interaction terms were removed from the model if they were not statistically significant until we arrived at the final model (Table S4). Due to the factorial design of the experiment, the oxygen*turbidity interaction term was always included in the model, regardless of statistical significance. Since this experiment examined multiple stressors by crossing oxygen and turbidity levels, these two stressors are by nature of the design interacting. We repeated ANCOVAs for each sex separately due to the significant sex*population interaction term. We tested all assumptions for parametric tests and the appropriateness of LMMs and ANCOVAs was confirmed. We used a difference in detection threshold between Trials 1 and 2 of ≥20 NTU as cut-off for inclusion in the rearing experiment analysis (Fig. S2) (i.e. any fish that had ≥20 NTU difference between clockwise and counterclockwise trials was excluded from the analysis). This threshold was higher in the lab compared to the field trials due to the difference in type of turbidity used and the relatively bright light in the laboratory environment. R software was used for all analyses (R Core Team, 2024), all ANCOVAs were performed using *stats* and all LMMs were performed using *Lme4*.

## Results

### Wild-caught fish

**Figure 1:**
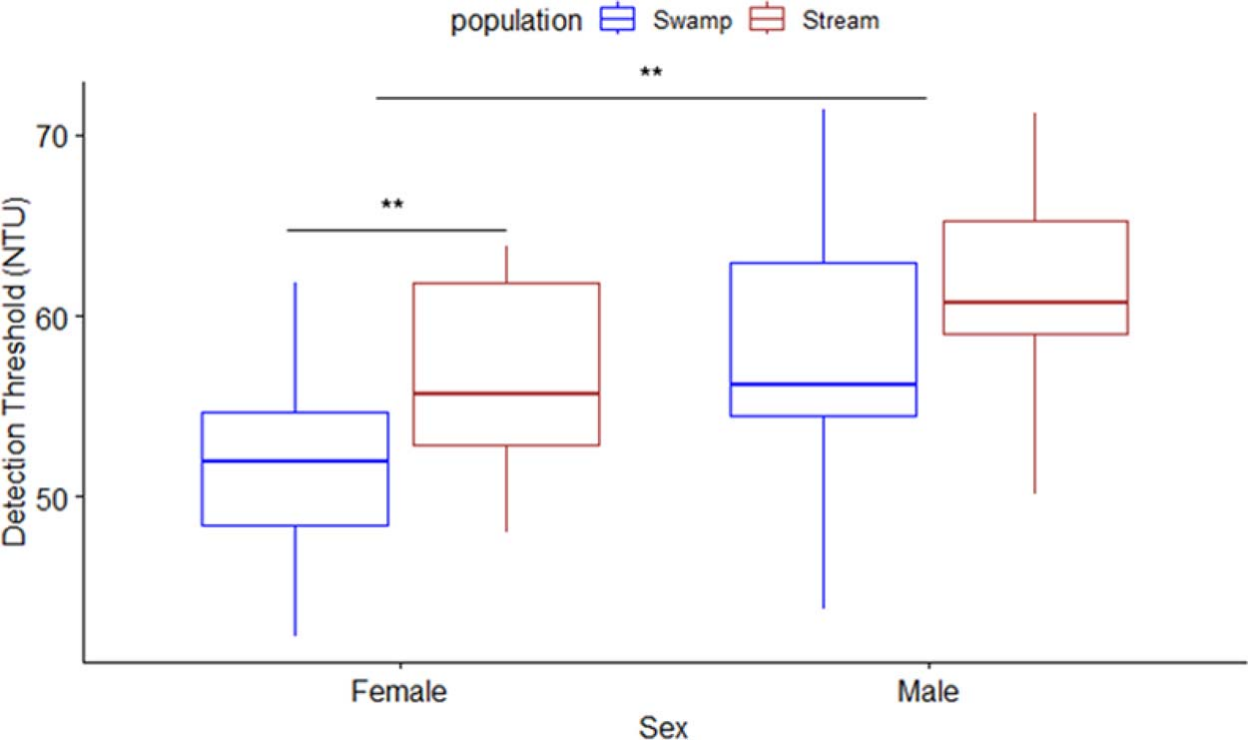
Boxplots of visual detection thresholds (turbidity level; NTU) for wild-caught *P. multicolor* (*n* = 35) from two populations (Female: *n* = 19; Male: *n* = 16; swamp: *n* = 18; river: *n* = 17) with significance indicators. Each individual was tested twice (once clockwise, once counterclockwise; order randomly determined), and the values were averaged. Boxplot displays the median and interquartile range with error bars.

We found that visual detection thresholds in wild-caught *P. multicolor* varied significantly by population (F_1,30_ = 5.715, *P* = 0.0233), sex (F_1,30_ = 3.885, *P* = 0.0580) and the interaction between population and standard length (F_1,30_ = 5.863, *P* = 0.0207), though standard length was not significant (Fig. 1, Table 1). The interaction between population and sex was not significant so this term was removed from the final model. This analysis revealed that wild-caught males had a higher detection threshold than females, and the river population had a slightly higher detection threshold than the swamp population.

**Table 1:** Results of ANCOVAs on visual detection threshold in two populations (river and swamp) of wild-caught *P. multicolor*

	*df*	Sum Sq	Mean Sq	F-value	*P-*value^a^
Population	1	236.6	236.6	5.715	0.0233^**^
Sex	1	160.8	160.8	3.885	0.0580^*^
log10(standard length)	1	0.001	0.006	0.0001	0.9901
Population*log10(standard length)	1	269.1	269.1	5.863	0.0207^**^

a ^a^Levels of significance: ^*^P < 0.1, ^**^P < 0.05.

Since *P. multicolor* is a sexually dimorphic species and detection thresholds varied between sexes in the main model for wild-caught fish, we also analysed females and males separately. In females, we found that detection thresholds varied significantly by population (F_1,15_ = 5.044, *P* = 0.0402) and the interaction of population and log10(standard length) (F_1,29_ = 6.343, *P* = 0.0236), such that females from the river site had higher detection thresholds than females from the swamp site (Fig. 1, Table 2A). Log10(standard length) was not statistically significant in explaining the observed variation. We found no differences in detection thresholds for males between populations or with standard length (Fig. 1, Table 2B).

**Table 2:** Results of ANCOVAs on visual detection threshold in two populations (river, swamp) of wild-caught *P. multicolor* separated by sex (females, males)

(A) Females					
	*df*	Sum Sq	Mean Sq	F-value	*P*-value^a^
Population	1	122.2	122.2	5.044	0.0402^**^
log10(standard length)	1	17.9	17.94	0.740	0.4031
Population*log10(standard length)	1	153.7	153.7	6.343	0.0236^**^
(B) Males					
	*df*	Sum Sq	Mean Sq	F-value	Pr(>F)
Population	1	45.1	45.06	0.854	0.374
log10(standard length)	1	7.5	7.5	0.142	0.713
Population*log10(standard length)	1	128.7	128.7	2.439	0.144

a ^a^Levels of significance: ^*^P < 0.1, ^**^P < 0.05.

### Rearing experiment

**Figure 2:**
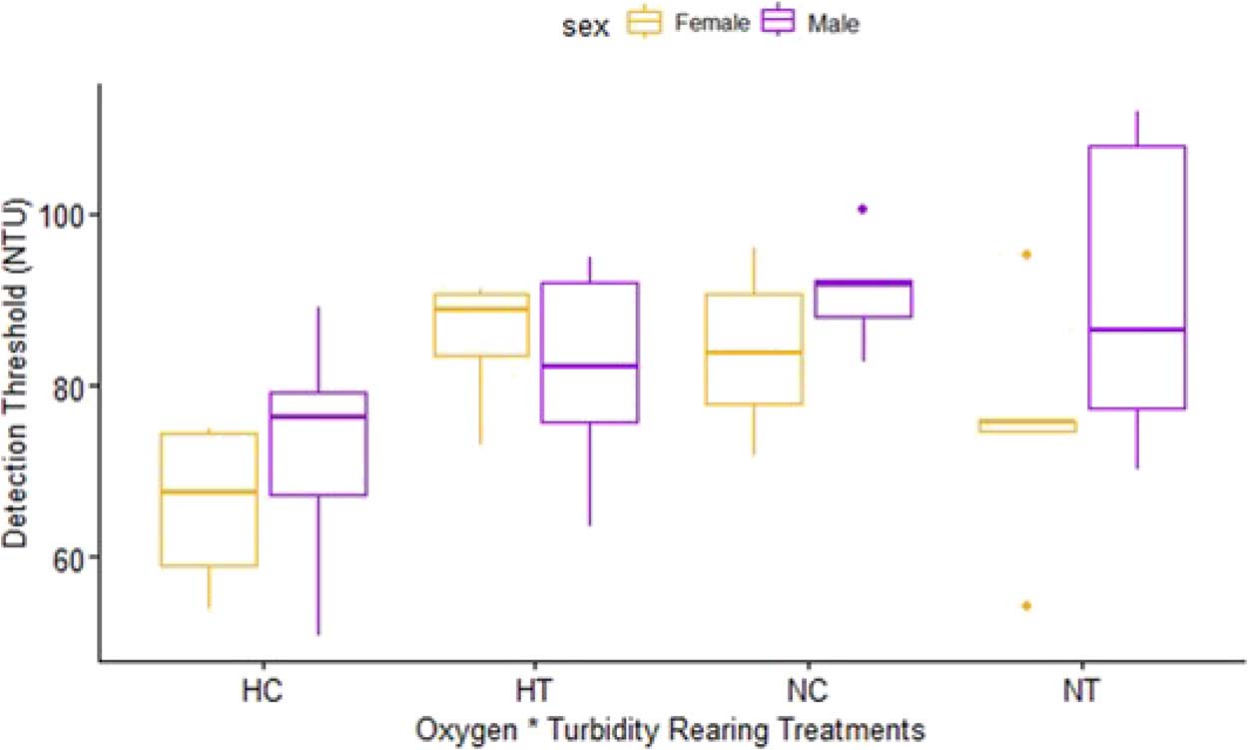
Boxplots of visual detection thresholds (turbidity level; NTU) for *P. multicolor* from the fully factorial split-brood rearing experiment (hypoxic-clear (HC): *n* = 12; hypoxic-turbid (HT): *n* = 12; normoxic-clear (HC): *n* = 12; normoxic-turbid (HT): *n* = 12). Each individual was tested twice (once clockwise, once counterclockwise; order randomly determined), and the values were averaged. Boxplot displays the median and interquartile range with error bars.

The final model that best described the variation in optomotor response in rearing experiment fish (i.e. F1 fish) was detection threshold ~ sex + population + turbidity + oxygen + log10(standard length) + sex*population + oxygen*turbidity (Table 3). We found that the oxygen rearing treatment (F_1,33_ = 5.608, *P* = 0.0239) significantly explained variation in detection thresholds of rearing experiment *P. multicolor,* but the turbidity rearing treatment alone was not significant (F_1,33_ = 1.498, *P* = 0.2297). Both the sex*population interaction term (F_1,33_ = 3.877, *P* = 0.0574) and the turbidity*oxygen interaction term (F_1,33_ = 3.260, *P* = 0.0801) approached significance in explaining the observed variation in detection thresholds and were therefore kept in the final model (Fig. 2). Population (F_1,33_ = 0.5732, *P* = 0.4543), sex (F_1,33_ = 1.822, *P* = 0.1863) and log10(standard length) (F_1,33_ = 1.092, *P* = 0.3036) were not significant. To account for potential maternal effect, brood was included as a random effect in a separate LMM. This model showed that brood was not statistically significant, thus brood was therefore removed from the analysis.

**Table 3:** Results of ANCOVAs on visual detection threshold in *P. multicolor* reared in a fully factorial split-brood rearing experiment (turbidity: clear vs turbid; oxygen: normoxia vs hypoxia)

	*df*	Sum Sq	Mean Sq	F-value	*P*-value^a^
Sex	1	272.6	272.6	1.8219	0.1863
Population	1	85.8	85.8	0.5732	0.4543
Turbidity	1	224.2	224.2	1.4979	0.2297
Oxygen	1	839.3	839.3	5.6084	0.0239^**^
log10(standard length)	1	163.4	163.4	1.0921	0.3036
Sex*population	1	580.2	580.2	3.8769	0.0574^*^
Turbidity*oxygen	1	487.8	487.8	3.2596	0.0801^*^

a ^a^Levels of significance: ^*^P < 0.1, ^**^P < 0.05.

Since *P. multicolor* is a sexually dimorphic species and because of the near-significant interaction term of sex and population, both F1 males and F1 females were analysed separately. When separating the models by sex, we observed that the turbidity*oxygen term approached significance for F1 females (F_1,33_ = 3.570, *P* = 0.0833). All other terms from the model were not significant (Table 4A). For males, the analysis revealed that oxygen was significant in explaining the variation in detection threshold (F_1,17_ = 4.606, *P* = 0.0466), and population approached significance (F_1,17_ = 3.709, *P* = 0.0710). No other terms were significant (Table 4B).

**Table 4:** Results of ANCOVAs on visual detection threshold in *P. multicolor* reared in a fully factorial split-brood rearing experiment (turbidity: clear vs turbid; oxygen: normoxia vs hypoxia) separated by sex (females, males)

(A) Females					
	*df*	Sum Sq	Mean Sq	F-value	*P*-value^a^
Population	1	212.8	212.8	1.4900	0.2457
Turbidity	1	38.97	38.97	0.2728	0.6120
Oxygen	1	66.74	66.74	0.4671	0.5073
log10(standard length)	1	220.38	220.38	1.5426	0.2380
Turbidity*oxygen	1	509.96	509.96	3.5696	0.0833^*^
(B) Males					
	*df*	Sum Sq	Mean Sq	F-value	*P*-value^a^
Population	1	639.61	639.61	3.7093	0.0710^*^
Turbidity	1	103.63	103.63	0.6010	0.4489
Oxygen	1	794.25	794.25	4.6061	0.0466^**^
log10(standard length)	1	0.74	0.74	0.0043	0.9485
Turbidity*oxygen	1	86.03	86.03	0.4989	0.4896

a ^a^Levels of significance: ^*^P < 0.1, ^**^P < 0.05.

## Discussion

We found that visual detection thresholds under increasing turbidity varied significantly depending on population of origin and sex for wild-caught *P. multicolor* (Fig. 1). Specifically, wild-caught females from the river population had higher detection thresholds, indicating that they could see better under elevated turbidity than females from the swamp; however, this trend did not hold for wild-caught males. We also found a significant interaction between population and standard length in wild-caught fish such that, on average, fish from the river population were 6.5% longer than the swamp population. This matches documented size differences between populations of wild-caught *P. multicolor* from swamp and river habitats (Chapman *et al.,* 2000). The results from our rearing experiment were less clear; on average, males reared under normoxic conditions had a ~15% higher detection threshold (i.e. could see better at higher turbidity) than males reared under hypoxic conditions. Surprisingly, females reared in hypoxic-turbid conditions had similar detection thresholds to those reared in normoxic-clear conditions, and on average, their detection threshold was ~23% higher than females reared in hypoxic-clear conditions and ~12% higher than females reared in normoxic-turbid conditions. The complexity of these sex-dependent responses to an altered visual landscape in the field and laboratory are explored below.

As predicted, we found a difference in detection threshold between wild-caught populations such that river fish had on average ~8% higher detection thresholds (mean: 59 NTU; i.e. could detect the difference between objects at a higher level of turbidity) than swamp fish (mean: 54 NTU), suggesting that exposure to chronic turbidity facilitates better vision under turbid conditions. While the difference was only about one step (~4NTU) in our optomotor trials, visual adaptations in the river population (e.g. larger eyes), may give them a slight advantage under turbid conditions resulting in the ability to detect contrast at higher turbidity levels than their swamp counterparts. Reduction in the amount of light and change in spectral composition associated with turbidity could favour larger eyes to maintain visual detection thresholds (Huber and Rylander, 1992; Huber *et al.,* 1997). Dugas and Franssen (2012) found that red shiner (*Cyprinella lutrensis*) from turbid sites had relatively larger eyes than fish from less turbid sites. River fish were larger than swamp fish (Table 1), and this could result in larger visual morphology (e.g. larger eyes, larger brain) explaining the higher detection thresholds. Larger eyes collect more light and tend to be correlated with higher visual acuity compared to smaller eyes (Caves *et al.,* 2017); it is possible that the documented differences in size between these two divergent populations influenced the detection thresholds observed here. However, we detected no relationship between standard length and detection threshold independent of population. Additionally, *P. multicolor* is a sexually dimorphic species, and males are substantially larger than females. Wild-caught males did have higher detection thresholds than females, and, indeed, this could be explained by size, and subsequently eye size (assuming an allometric relationship), playing a role in visual detection abilities (Tiarks, 2024).

When we assessed wild-caught fish by sex, we found that, for females, population and the interaction of population and size were significant in explaining the observed variation in detection threshold. Females originating from the turbid river environment had ~9% higher detection thresholds compared to their clear swamp environment counterparts; females from the river population could see in water up to 57 NTU (on average), while females from the swamp environment stopped detecting contrast at 52 NTU (on average). Ambient environmental light is a strong ecological driver that shapes visual sensitivity, such that species tune their visual systems towards the spectrum of light present in their environment (Parry *et al.,* 2005; Carleton *et al.,* 2008; Seehausen *et al.,* 2008; Carleton *et al.,* 2020). While habitat light conditions may drive ecological selection, body coloration and contrast with the ambient environment likely contribute to visual system tuning in sexually dimorphic cichlids via sexual selection. Research points to wavelength-shifted visual sensitivity in cichlids to match light habitat, and this concurrently evolves with nuptial coloration to correspond to their visual sensitivity (e.g. Seehausen *et al.,* 2008; Schneider *et al.,* 2020). Our results suggest that females from turbid environments are able to detect contrast at higher levels of turbidity compared to females from swamp environments, and this ability could help them to distinguish nuptial displays (e.g. mating displays, coloration) in the turbid environments they are found in. Female mate choice is an important factor driving population persistence in African cichlids, and our results suggest that there is a population-specific component maintaining the differences in observed detection thresholds for females from different populations.

We predicted an antagonistic response to multiple stressors in the rearing experiment: we predict that fish reared in high turbidity would have better vision, while fish reared in low DO would have worse vision due to energetic costs associated with vision. When these stressors were combined (i.e. hypoxic-turbid), we predicted that the resulting effect would result in worse vision than either of the stressors on their own. Surprisingly for females, we observed a synergistic response in an unexpected way. We predicted that the most stressful environment (hypoxic-turbid) would have the lowest detection thresholds, yet we observed the highest detection threshold (i.e. better vision) in the most stressful environment. It is possible that there was an energetic trade-off/investment into visual physiology that could explain the increased visual detection threshold observed in female fish reared in the hypoxic-turbid treatment. One explanation is that the response to multiple stressors was to increase investment in bigger eyes; however, hypoxia alone was not enough to trigger investment into the visual system (at least not enough to result in higher detection thresholds). For males, oxygen as an individual stressor appears to have acted as an energy constraint, and this could explain the lower detection thresholds observed in hypoxic rearing conditions. Future research should directly test the energetic trade-off of investment in the visual sensory system. Additionally, further research should investigate direct links between behavioural modifications and physiological and/or morphological responses to environmental fluctuations. We recognize that there was a relatively small sample size for the rearing experiment fish. Some of the hypoxic treatments for the lab-reared fish had low sample sizes due to the stressful nature of the low-oxygen-rearing treatment that impacted the number of fish available for the optomotor behavioural assay; regardless, the results indicate trends that should not be overlooked nor overstated.

A key difference we observed from wild-caught fish versus those reared in the laboratory under controlled settings was the mean detection threshold. Detection threshold in the laboratory averaged 81 NTU, while it was lower in the field, averaging 57 NTU. A combination of things could explain this difference, in particular the ambient light environment and turbidity composition used during trials. Optomotor response trials were conducted under indirect natural sunlight (i.e. full-spectrum light) in the field, while trials in the lab were conducted under broad-spectrum overhead lighting. The broad-spectrum overhead lights in the laboratory (i.e. only a small fraction of UV light) may have resulted in different visual response in rearing experiment fish relative to wild-caught fish where the trials were conducted outside. Brightness also varied between the field and the lab, such that the lab environment had much brighter light than the natural lighting in the field. Additionally, detection threshold responses are expected to vary depending on the composition of particulate types found in areas of elevated turbidity (Gray, 2016). The composition and concentrations of particulate matter inherently affects how light is scattered and absorbed when it enters a water column (Lythgoe, 1980). Optomotor response trials in the field used sediment from the field sites to accurately represent the turbidity experienced by wild populations. In the lab, a bentonite clay sediment was used because of its ability to stay evenly suspended; however, the bentonite clay had a grey hue and is more uniform in shape and size, while the field turbidity had an orange hue and is more variable in shape and size. Therefore, there were spectrum, brightness and colour differences between lab and field trials that likely explain some of the variation seen between lab and field trials. This suggests that patterns for wild-caught fish, both absolute and relative, may differ from those found in the laboratory environment.

Behavioural adjustments are typically the first way an organism can respond to fluctuation in environmental conditions (Wong and Candolin, 2015). Our results suggest that turbidity affects behaviourally expressed visual sensitivity, and that this could potentially affect visually mediated behaviours, especially behaviours reliant on contrast detection. It is important to note that there are additional components that could affect visual sensitivity, such as opsin gene expression (e.g. Carleton *et al.,* 2016) or visual morphological differences (e.g. Tiarks *et al.,* 2024), that could help maintain these observed behavioural differences. We might expect physiological mechanisms to maintain the difference in behaviourally expressed detection threshold between male and female fish (as we saw in our results), but the underlying mechanisms might not be so clear. For example, a recent study by Schneider *et al.* (2020) found no differences in photic visual sensitivity or opsin gene expression in sexually dimorphic cichlids. Future studies should examine the specific drivers maintaining and facilitating behavioural adjustments, like higher detection thresholds, to changes in the visual environment.

## Supplementary Material

Web_Material_coaf046

## Data Availability

All data are available on Dryad: https://datadryad.org/dataset/doi:10.5061/dryad.3j9kd51x3
