# Supplementary material for: Sex-dependent shifts in visual detection thresholds under turbid conditions in an African cichlid
Source: Conserv Physiol. 2025 Jul 14;13(1):coaf046. doi: 10.1093/conphys/coaf046 (PMC12256141; doi:10.1093/conphys/coaf046)
Supplement: Web_Material_coaf046 [file web_material_coaf046.pdf]

Supplemental Material for: Sex-dependent shifts in visual detection thresholds under turbid conditions in an African cichlid

J.H. Tiarks and S.M. Gray

Table S1: Raw biometric data of two wild caught populations (river, swamp) of *Pseudocrenilabrus multicolor* divided by sex.

Population	Sex	N	Mass (g) ± se	Standard Length (cm) ± se
River (NYA)	F	9	1.08 ± 0.07	2.97 ± 0.08
	M	8	2.01 ± 0.20	3.83 ± 0.19
Swamp (LWA)	F	10	0.90 ± 0.06	2.79 ± 0.07
	M	8	1.80 ± 0.24	3.60 ± 0.18

Table S2: Raw biometric data for *Pseudocrenilabrus multicolor* used in the rearing experiment (NC: normoxic clear; NT: normoxic turbid; HC: hypoxic clear; HT: hypoxic turbid) divided by sex.

Rearing	Sex	N	Mass (g) ± se	Standard Length (cm) ± se
HC	F	4	1.46 ± 0.12	3.55 ± 0.03
	M	7	2.00 ± 0.22	3.87 ± 0.14
HT	F	4	1.74 ± 0.19	3.58 ± 0.13
	M	6	2.52 ± 0.36	4.12 ± 0.22
NC	F	5	2.28 ± 0.19	3.96 ± 0.13
	M	5	3.87 ± 0.33	5.02 ± 0.15
NT	F	5	1.64 ± 0.15	3.60 ± 0.11
	M	5	4.59 ± 0.64	5.28 ± 0.22

Table S3: AIC ranking table for model selection for wild caught fish.

Model	AIC value
DT ~ sex + population + log10(SL) + sex*population + sex*log10(SL) + population*log10(SL) + sex*population*log10(SL)	241.75
DT ~ sex + population + log10(SL) + sex*population + sex*log10(SL) + population*log10(SL)	239.25
DT ~ sex + population + log10(SL) + sex*population + population*log10(SL)	238.64
**DT ~ sex + population + log10(SL) + population*log10(SL)**	**236.03**

Table S4: AIC ranking table for model selection for reared fish.

Model	AIC value
DT ~ sex + population + turbidity + oxygen + log10(SL) + sex*population + sex*turbidity + population*turbidity + sex*oxygen + population*oxygen + turbidity*oxygen + sex*population*turbidity*oxygen	341.2993
DT ~ sex + population + turbidity + oxygen + log10(SL) + sex*population + sex*turbidity + population*turbidity + sex*oxygen + population*oxygen + turbidity*oxygen	337.5519
DT ~ sex + population + turbidity + oxygen + log10(SL) + sex*population + sex*turbidity + sex*oxygen + population*oxygen + turbidity*oxygen	335.7286
DT ~ sex + population + turbidity + oxygen + log10(SL) + sex*population + sex*oxygen + population*oxygen + turbidity*oxygen	333.903
DT ~ sex + population + turbidity + oxygen + log10(SL) + sex*population + population*oxygen + turbidity*oxygen	332.4901
**DT ~ sex + population + turbidity + oxygen + log10(SL) + sex*population + turbidity*oxygen**	**330.7924**

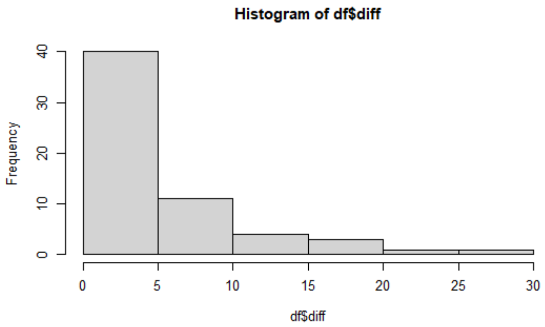

Figure S1: Histogram of the difference between detection thresholds in Trial 1 and Trial 2 for wild-caught fish. Individuals with a difference ≥10NTU were excluded from analysis; this ensured that we observed a consistent, repeatable detection threshold as determined in this optomotor behavioral assay. Additionally, this removed outliers and captured the majority of observations.

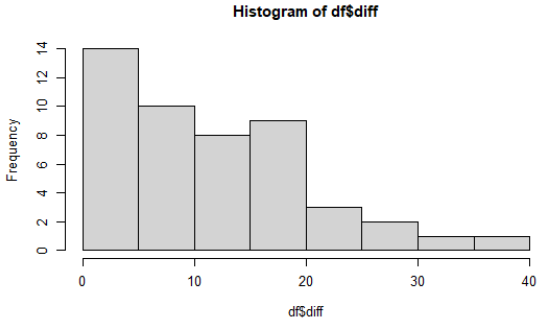

Figure S2: Histogram of the difference between detection thresholds in Trial 1 and Trial 2 for F1 fish used in the rearing experiment. Individuals with a difference ≥20NTU were excluded from analysis; this ensured that we observed a consistent, repeatable detection threshold as determined in this optomotor behavioral assay. Additionally, this removed outliers and captured the majority of observations.
